# Safety of Adalimumab and Predictors of Adverse Events in 1693 Japanese Patients with Crohn’s Disease

**DOI:** 10.1093/ecco-jcc/jjw060

**Published:** 2016-03-09

**Authors:** Haruhiko Ogata, Mamoru Watanabe, Toshiyuki Matsui, Hidenori Hase, Motohiro Okayasu, Tsuyoshi Tsuchiya, Yasuhiko Shinmura, Toshifumi Hibi

**Affiliations:** ^a^Center for Diagnostic and Therapeutic Endoscopy, School of Medicine, Keio University, Tokyo, Japan; ^b^Department of Gastroenterology and Hepatology, Graduate School, Tokyo Medical and Dental University, Tokyo, Japan; ^c^Department of Gastroenterology, Fukuoka University Chikushi Hospital, Fukuoka, Japan; ^d^Medical Division at AbbVie G.K., Tokyo, Japan; ^e^Center for Advanced IBD Research and Treatment, Kitasato Institute Hospital Kitasato University, Tokyo, Japan

**Keywords:** Adalimumab, Crohn’s disease, post-marketing study

## Abstract

**Background and Aims:**

Data from an all-cases post-marketing study were used to evaluate the safety and effectiveness of adalimumab in Japanese patients with Crohn’s disease [CD].

**Methods:**

Patients received adalimumab for 24 weeks. Data from all patients [*n* = 1693] were used for the safety assessment. Data from patients with CD activity index [CDAI] ≥ 150 at baseline were used for the effectiveness assessment.

**Results:**

The most frequent serious adverse drug reaction [ADR] was infection and infestations [6.6 events/100 patient-years]. The risk of serious infections increased in patients who had a history of malignancy and those with concomitant corticosteroid use. Of 415 patients who had switched from another anti-tumour necrosis factor alpha [TNFα] agent to adalimumab due to ADRs, 7.2% discontinued due to ADRs to adalimumab. Ten of 13 patients with a history of tuberculosis [TB] received prophylactic medication, and none developed TB. TB developed in one patient with no history of TB or anti-TB prophylaxis. Remission rates were 41.3% and 32.4% at 4 and 24 weeks, respectively. Remission rates did not differ between patients with and without concomitant use of immunomodulators. Predictive variables for increased effectiveness were CDAI ≤ 220 and disease duration of ≤ 2 years. Perianal lesions and loss of response to previous anti-TNFα agents affected effectiveness.

**Conclusions:**

The most frequent serious ADR was infection. Adalimumab significantly reduced disease activity, without any unexpected ADRs. Development of active TB during adalimumab therapy can be prevented through TB screening and prophylaxis. In patients who switched from another anti-TNFα agent to adalimumab due to ADRs, adalimumab was well tolerated.

## 1. Introduction

Crohn’s disease [CD] is a chronic inflammatory bowel disease [IBD] characterised by relapse and remission, with progression over time to the complications of stricture, fistulas, or abscesses.^1^ Surgery may be required for the treatment of persistent intestinal obstruction, abscess, or fistula.

Antibodies to the proinflammatory cytokine tumour necrosis factor alpha [TNFα] can be effective in improving symptoms and inducing remission of CD,^2^ and have shown promise in improving quality of life and decreasing rates of hospitalisation and surgery. The currently available anti-TNFα agents for the treatment of CD in Japan include infliximab [Remicade; Janssen Biotech Inc., Malvern, PA, USA]^3^ and adalimumab [Humira; AbbVie Inc., North Chicago, IL, USA].^4^

Adalimumab, a fully human anti-TNFα monoclonal antibody, has been shown to be effective and generally well tolerated when used for both induction and maintenance therapy for CD.^5^ In addition, the sustained benefits of long-term use of adalimumab in clinical practice have been confirmed by the results of both Western^6^ and Japanese studies.^7^ However, evidence from large-scale population-based studies involving Japanese patients receiving adalimumab in clinical practice remains insufficient.

In the present study, we used data from a post-marketing study for all patients receiving adalimumab for the treatment of CD, after the approval of the disease as an indication for adalimumab, to evaluate the safety [the assessment included identification of any unknown adverse drug reactions, ADRs] and effectiveness of adalimumab in clinical practice in Japanese patients with active CD. In addition, factors affecting the safety and effectiveness of adalimumab were investigated.

## 2. Methods

### 2.1. Study design

This multicentre single-cohort non-interventional observational study was performed to evaluate the safety and effectiveness of adalimumab in patients with active CD, who were enrolled between October 27, 2010 and October 29, 2012 from 802 medical institutions in Japan, and followed for 24 weeks in the clinical setting. Information regarding unknown ADRs and factors that might affect the safety and effectiveness of adalimumab was also collected. The study protocol was submitted to and approved by the Pharmaceuticals and Medical Devices Agency in Japan before the study began. This study was conducted in registered medical institutions in compliance with Good Post-marketing Study Practice in Japan, and is in the ClinicalTrials.gov registry [NCT01298648].

### 2.2. Patients

In accordance with the current list of indications in the package insert for adalimumab in Japan, patients with moderate to severe active CD and not responding well to conventional therapies were suitable candidates for adalimumab treatment and therefore eligible for the study. Patients received subcutaneous injections of adalimumab every other week at the starting dose of 160mg, followed by a subsequent dose of 80mg at Week 2, and 40mg every other week thereafter for 24 weeks.

### 2.3. Safety assessment

In the present study, data on all adverse events [AEs] reported in patients enrolled in the study were used in the safety analysis. The assessment of safety included the evaluation of AEs up to Week 24. AEs were classified by system organ class [SOC] and coded with preferred terms using the bilingual [English‒Japanese] version of the Medical Dictionary for Regulatory Activities [MedDRA/J], version 15.0. Text data for concomitant drugs were coded in accordance with the Japanese local drug dictionary [Saishinsa Iyakuhin Code List] for statistical analyses. Groupings of these data were the same as those used in clinical trials required for drug approval in Japan. AEs for which causality with adalimumab could not be ruled out were defined as ADRs. Assessments were made for all AEs, serious AEs, ADRs, serious ADRs, infections, and serious infections. Univariable analysis and multivariable analysis were performed to determine factors associated with serious ADRs and serious infections. The Wald test was used to calculate odds ratios and their 95% confidence intervals.

### 2.4. Effectiveness assessment

The effectiveness analysis set consisted of data from patients with a baseline CD activity index [CDAI] score of ≥ 150. CDAI was evaluated at baseline and at Weeks 4 and 24. Reduction of CDAI to < 150 from baseline was considered as achievement of clinical remission. A logistic regression model was used to identify potential predictive variables for effectiveness. Factors associated with clinical remission at Week 24 were investigated by multivariate analysis using the following subgroups: baseline CDAI [150‒220, 220‒300], disease duration [< 2 vs < 5 years, < 2 vs < 10 years, < 2 vs < 20 years, and < 2 vs ≥ 20 years], perianal lesions, and loss of response [LOR] to previous anti-TNFα agents.

### 2.5. Statistical analysis

The collected observational data were used for the analysis of effectiveness. Clinical remission rates were analysed by both non-responder imputation [NRI] and as-observed analyses. Some patients did not complete the 24-week study, so missing data on clinical remission rates at each time point were analysed using NRI and as-observed analyses, in which missing values were counted as non-responders and uncountable, respectively. A logistic regression model was used to identify potential predictive variables for the achievement of clinical remission at Week 24, as well as for serious ADRs and serious infections. A stepwise selection procedure was used to select factors for inclusion in the final model [with the criterion of *p*-value 0.10 for entry and 0.05 for remaining in the model].

The chi-square test was used for all statistical comparisons. Within each disease duration subgroup, data from patients achieving remission at Weeks 4 and 24 were analysed by using the Cochran‒Armitage test. All tests were two-sided and done by using SAS System Release 9.1 [SAS Institute Inc., Cary, NC, USA], with *p* < 0.05 defined as significant.

## 3. Results

### 3.1. Baseline characteristics

**Figure 1.**
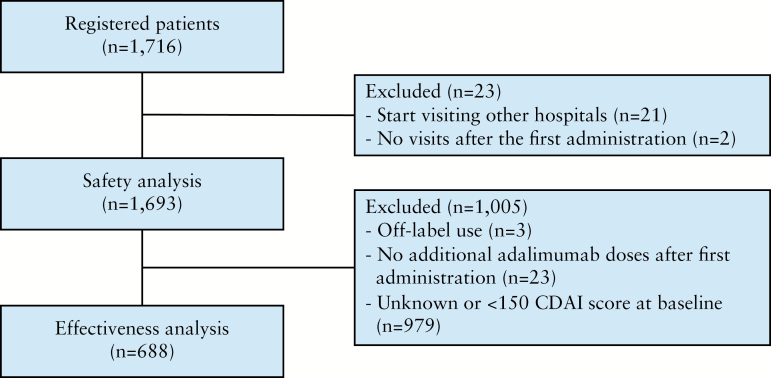
Patient disposition. CDAI, Crohn’s disease activity index.

A total of 1716 patients were enrolled between October 27, 2010 and October 29, 2012. After the exclusion of 21 patients who started visiting other hospitals during the observational period and 2 patients who did not visit the hospital after the first administration of adalimumab, the safety analysis set consisted of data from 1693 patients. Of these patients, those who received off-label treatment with adalimumab [for Behcet’s disease in two patients, and chronic non-specific ulcer of the small intestine in one patient], those who did not receive any additional adalimumab doses after the first administration, and those whose CDAI score was unknown or < 150 at baseline were excluded, so the effectiveness analysis set consisted of data from 688 patients [Figure 1].

Table 1 shows the baseline characteristics of the 1693 patients whose data were used in the safety analysis. The male-to-female ratio was about 2:1, and the mean age was 35.5 years. The mean duration of CD was 11.1 years; most patients [30.5] had had CD for 10‒20 years; 22.3% had had it for 5‒10 years; and 23.3% for < 5 years. Mean baseline CDAI score was 204.6. Most patients [91.8%] were receiving concomitant treatment with adalimumab therapy: 84.9% with aminosalicylates, 18.9% with corticosteroids, 31.1% with immunomodulators [IMs], and 11.6% with antimicrobials. Crohn’s disease-related intestinal complications [eg stenosis, fistula, perforation, and adhesion] and extraintestinal complications [eg iritis, aphthous stomatitis, and erythema nodosum] were present in 61.1% and 17.5% of patients, respectively. About half of patients [51.6%] had a history of surgery for the treatment of CD.

**Table 1.** Baseline demographics and clinical characteristics.

Sex, *n* [%]	
Male	1109 [65.5]
Female	584 [34.5]
Age [years]	35.5±11.7^a^
Body weight [kg]	54.7±10.8^a^
Duration of CD [years]	11.1±8.0^a^
CDAI score^b^	204.6±106.5^a^
Pretreatment drugs, *n* [%]	
Anti-TNFα agent	1306 [77.1]
Aminosalicylates	1497 [88.4]
Corticosteroids	463 [27.3]
Immunomodulators	580 [34.3]
CD-related antibiotics	305 [18.0]
Others	7 [0.4]
Concomitant drugs, *n* [%]	
Aminosalicylates	1437 [84.9]
Corticosteroids	320 [18.9]
Immunomodulators	526 [31.1]
Antimicrobials for CD treatment	196 [11.6]
Concomitant therapy, *n* [%]	
Granulocyte adsorption apheresis	10 [0.6]
Enteral nutrition therapy	941 [55.6]
Intravenous nutrition therapy	124 [7.3]
Others	45 [2.7]
History of surgical operation, *n* [%]	
No	819 [48.4]
Location of CD lesions, *n* [%]	
Small bowel	154 [9.1]
Colon	232 [13.7]
Small bowel and colon	421 [24.9]
Other	12 [0.7]
Location of CD lesions,^c^, *n*[%]	
Perianal lesions	824 [48.7]
Rectal	599 [35.4]
Gastroduodenal	163 [9.6]
Colon	1157 [68.3]
Jejunum	251 [14.8]
Ileum	1258 [74.3]
Other	45 [2.7]
Complications, *n* [%]	
Liver disorders	72 [4.3]
Renal disorders	31 [1.8]
Circulatory disorders	44 [2.6]
Blood disorders	231 [13.6]
Respiratory disorders	49 [2.9]
Other	510 [30.1]
Crohn’s disease-related intestinal complications, *n* [%]	
No	656 [38.7]
Yes	1035 [61.1]
Crohn’s disease-related extraintestinal complications, *n* [%]	
No	1394 [82.3]
Yes	297 [17.5]

CD, Crohn’s disease; CDAI, Crohn’s Disease Activity Index; TNF, tumour necrosis factor.

^a^Mean ± standard deviation [SD].

^b^Data in the effectiveness analysis set [*n* = 688].

^c^Data include both current and previous disease history.

At baseline, 4 patients had malignancy [chronic myelogenous leukaemia, acute myelogenous leukaemia, breast cancer, and ovarian cancer], and 11 patients had a history of malignancy [gastric cancer, malignant lymphoma, rectal cancer, and bladder cancer in two patients each, and breast cancer, renal cell carcinoma, and cervical cancer in one patient each].

Most patients had received previous anti-TNFα therapy [77.1%]. Table 2 summarises the reasons for its discontinuation; 55.7% of patients had LOR to anti-TNFα therapy. For 136 patients with other reasons for discontinuation, these were patient-related matters, physician’s decision, completion of the clinical study of adalimumab, and unknown reasons.

**Table 2.** Previous anti-TNFα exposure and reason for discontinuation

Prior anti-TNFα exposure, *n* [%]	
No	387 [22.9]
Yes	1306 [77.1]
Intolerance of former anti-TNFα therapy	72/1306 [5.5]
Loss of response to former anti-TNFα therapy	727/1306 [55.7]
Acute AEs	306/1306 [23.4]
Delayed AEs	100/1306 [7.7]
Others	136/1306 [10.4]

AE, adverse event; TNF, tumour necrosis factor.

### 3.2. Safety

The overall incidence rates for ADRs and serious ADRs were 76.1/100 patient-years [PYs], and18.3/100 PYs, respectively. By MedDRA SOC definitions, ‘infections and infestations’ [19.9/100 PYs] was the most frequent ADR category, followed by ‘general disorders and administration-site conditions’ [14.3/100 PYs], ‘gastrointestinal disorders’ [10.3/100 PYs], and ‘skin and subcutaneous tissue disorders’ [9.8/100 PYs]. The most frequent serious ADRs were ‘infections and infestations’ [6.6/100 PYs] and ‘gastrointestinal disorders’ [4.5/100 PYs] [Table 3]. There was no incidence of *Pneumocystis jiroveci* pneumonia, serious herpes infection [herpes zoster], fungus infection, or cytomegalovirus infection. Tuberculosis, sepsis, and herpes zoster were reported in 1 patient, 5 patients, and 11 patients, respectively.

**Table 3.** Adverse drug reactions by SOC classification.^a^

	Adverse drug reaction	Serious adverse drug reaction
Exposure, PYs	711.8	711.8
All	76.1	18.3
Infections and infestations	19.9	6.6
General disorders and administration-site conditions	14.3	0.8
Gastrointestinal disorders	10.3	4.5
Skin and subcutaneous tissue disorders	9.8	0.6
Investigations	3.8	0.7
Nervous system disorders	3.4	1.0
Respiratory, thoracic, and mediastinal disorders	3.0	0.3
Hepatobiliary disorders	2.5	0.3
Infections of interest		
Nasopharyngitis	2.5	
Pharyngitis	1.8	
Pneumonia^b^	1.8	1.3
Bronchitis	1.7	
Herpes zoster	1.4	0.4
Anal abscess	1.1	0.8
Sepsis^c^	0.8	0.8
Abdominal abscess	0.6	0.4
Gastrointestinal disorders of interest		
Ileus^d^	1.4	1.3
Intestinal obstruction	0.8	0.8

SOC, system organ class; PY, person-years.

^a^Events per 100 patient-years [PYs].

^b^Including *Pseudomonas* pneumonia and bacterial pneumonia.

^c^Including *Pseudomonas* sepsis.

^d^Including subileus disorders.

Screening tests for tuberculosis infection and hepatitis B virus were performed before the study treatment. Of the 1693 patients, 1435 patients [84.8%] underwent either tuberculin skin test/interferon-gamma release assays [IGRAs] or chest X-ray/computed tomography [CT] scan [Supplementary Table 1a, available as Supplementary data at *ECCO-JCC* online]. Of those who did not undergo tuberculin skin test or IGRAs, a chest X-ray or CT scan was done in 341 of 581 patients [58.7%] who had history of anti-TNFα therapy, and 44 of 62 [71.0%] who had no history of anti-TNFα therapy. Supplementary Table 2, available as Supplementary data at *ECCO-JCC* online, summarises the use of prophylactic medication by each inspection item. Of the 13 patients with a history of active tuberculosis, 10 patients [76.9%] received prophylaxis and 3 did not [2 patients had previously received a full course of therapy for tuberculosis, and 1 patient was shown by CT not to have active tuberculosis and refused prophylactic use of antitubercular drugs]. Of the patients with a positive tuberculin response, 107 patients [88.4%] did not receive preventive antituberculosis medication, but all three IGRA-positive patients did. The seven patients who had chest X-ray abnormalities and two patients who had chest CT scan abnormalities did not receive any preventive antituberculosis medication, because none of these patients had findings suggestive of obsolete pulmonary tuberculosis. Tuberculosis developed in one patient during adalimumab therapy; this patient had undergone screening tests including chest X-ray [normal], CT scan [normal], and tuberculin skin test [positive], but not IGRAs. He did not receive isonicotinic acid hydrazide before adalimumab therapy, because his chest X-ray and CT scan findings were normal, and no induration or double redness was detected by tuberculin skin test. Three months after the administration of adalimumab, he had a positive tuberculosis PCR test, as well as abnormal X-ray and CT scan findings in both upper lobes of the lung. Active pulmonary tuberculosis was diagnosed and successfully treated with a total of four antituberculosis agents for 3 months.

The hepatitis B antigen test was performed at baseline for 1404 of 1693 patients [82.9%] [Supplementary Table 1b]. De novo hepatitis B virus infection was found in one patient. Although he had tested negative for hepatitis B surface antigen before study treatment, hepatitis B surface antibody and hepatitis B core antibody tests were not performed. After he was found to have increased levels of hepatic transaminases, the results of tests for hepatitis B core antibody and hepatitis B virus DNA were found to be positive. The increased levels of transaminases had been found at the time of switching from previous anti-TNFα therapy to adalimumab. The patient discontinued adalimumab therapy and started to receive oral entecavir hydrate 0.5mg/day. He remained asymptomatic and had a negative test result for hepatitis B virus 4 months later. A total of 13 patients tested positive for hepatitis B surface antigen and received treatment with adalimumab; of whom 7 patients received treatment with entecavir and 6 patients received no treatment for hepatitis B virus infection. No patient had reactivation of hepatitis B virus or ADRs related to hepatic diseases.

In the present study, four patients had malignant disease at baseline but received adalimumab because of the absence of other therapeutic options. Of these patients, one patient with chronic myelocytic leukaemia had worsening of the disease, and recurrent leukaemia was reported as an ADR. However, the outcome was ‘improvement’. The other three patients had no ADRs related to malignancy.

During the study period, nine patients [0.5%] died; the causes of death were infections [two patients], hepatobiliary disorders [two patients], cardiac disorders [two patients], sudden death [one patient], peritonitis resulting from intestinal perforation [one patient], and haemorrhagic shock [one patient]. Causality with adalimumab could not be ruled out for the causes of death of two of the nine patients: sepsis [in one patient], and pneumonia and sepsis [in one patient] [data not shown].

The incidence of ADRs in a subgroup of patients who had a history of anti-TNFα therapy was also analysed. Of the 415 anti-TNFα‒treated patients, 118 patients [28.4%] had ADRs during treatment with adalimumab, and 28 patients [6.7%] had serious ADRs. Of the patients who discontinued adalimumab after switching from previous anti-TNFα therapy, ADRs were the reason for discontinuation for only 7.2%, therefore showing high tolerability of adalimumab [Table 4]. None of the ADRs that occurred differed from those previously reported,^5^ and no ADRs specific to adalimumab were identified.

**Table 4.** Adverse drug reactions and discontinuation of adalimumab in patients with a history of anti-TNFα therapy.

	Discontinuation of previous anti-TNFα therapy due to ADRs			Discontinuation of previous anti-TNFα therapy due to reasons other than ADRs [*n* = 885]	*p*-Value^a^
	Acute ADRs [*n* = 312]	Delayed ADRs [*n* = 109]	Total [*n* = 415]		
Discontinuation of adalimumab therapy, *n* [%]					
No	274 [87.8]	87 [79.8]	356 [85.8]	714 [80.7]	0.0245
Yes	38 [12.2]	22 [20.2]	59 [14.2]	171 [19.3]	
Discontinuation of adalimumab therapy due to ADRs, *n* [%]					
No	295 [94.6]	96 [88.1]	385 [92.8]	851 [96.2]	0.0085
Yes	17 [5.4]	13 [11.9]	30 [7.2]	34 [3.8]	
Incidence of ADRs, *n* [%]					
No	233 [74.7]	66 [60.6]	297 [71.6]	721 [81.5]	< 0.0001
Yes	79 [25.3]	43 [39.4]	118 [28.4]	164 [18.5]	
Incidence of serious ADRs, *n* [%]					
No	291 [93.3]	101 [92.7]	387 [93.3]	834 [94.2]	0.4886
Yes	21 [6.7]	8 [7.3]	28 [6.7]	51 [5.8]	

ADR, adverse drug reaction; TNF, tumour necrosis factor.

^a^Chi-square test.

**Figure 2.**
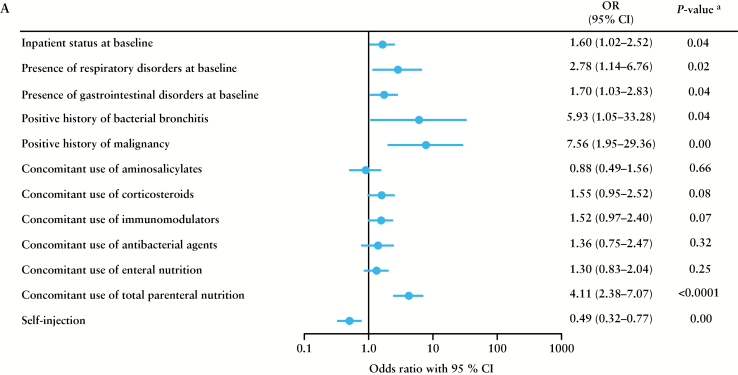

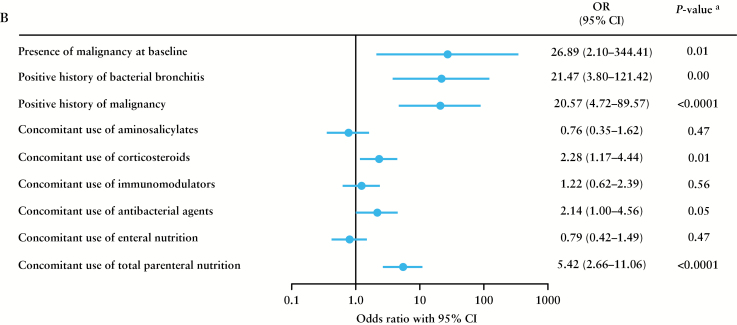
Factors affecting the safety of adalimumab identified by multiple logistic regression analysis for a] serious adverse drug reactions [ADRs] and b] serious infections. ^*a*^ *p*-value was calculated using Wald’s chi-square test. CI, confidence interval; OR, odds ratio.

The results of the multiple logistic regression analysis identified the following risk factors affecting the safety of adalimumab: inpatient status at baseline, respiratory disorders at baseline, gastrointestinal disorders at baseline, history of bacterial bronchitis, history of malignancy, concomitant use of total parenteral nutrition, and being the recipient of injections by medical personnel for serious ADRs [Figure 2a], malignancy at baseline, history of bacterial bronchitis, history of malignancy, concomitant use of corticosteroids, concomitant use of antibacterial agents, and concomitant use of total parenteral nutrition for serious infections [Figure 2b]. The risk of serious ADRs increased in patients receiving injections by medical personnel compared with self-injectors. The risk of serious infections increased in patients who had a history of malignancy and those with concomitant use of corticosteroids.

### 3.3. Effectiveness

**Figure 3.**
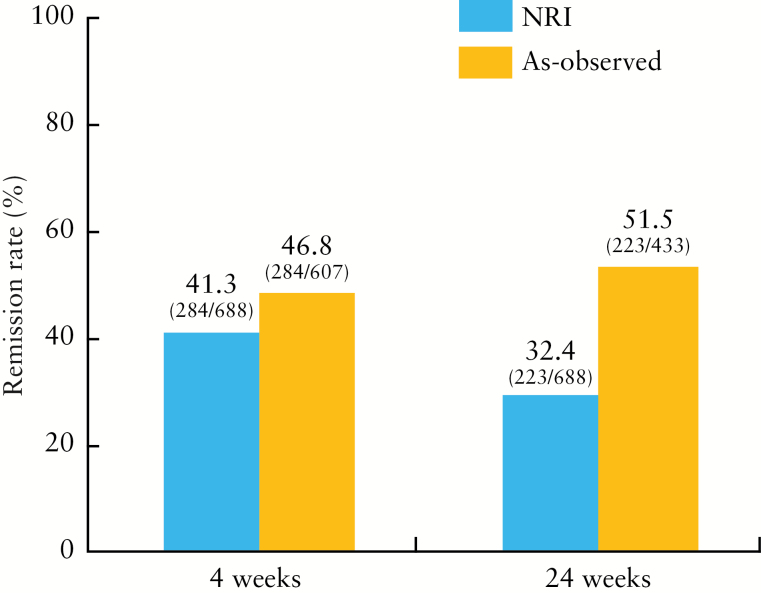
Patients achieving remission at 4 weeks and 24 weeks by non-responder imputation [NRI] and as-observed analyses.

The analysis of effectiveness used data from 688 patients with a CDAI score of ≥ 150 at baseline. Figure 3 shows remission rates after 4 and 24 weeks, based on NRI and as-observed analyses. In both analyses, clinical remission rates were > 40% at 4 weeks, whereas at 24 weeks the results varied [32.4% by NRI vs 51.5% by as-observed]. There was no significant difference in clinical remission rate between patients with and without concomitant IMs by NRI [41.2% and 41.3% at 4 weeks and 34.6% and 31.4% at 24 weeks] [Supplementary Figure 1, available as Supplementary data at *ECCO-JCC* online]. Analyses of subgroups stratified by disease duration using NRI and as-observed analyses were also performed. Clinical remission rates after 4 and 24 weeks were significantly higher in patients with shorter disease duration compared with those with longer duration [week 4, *p* < 0.0001; week 24, *p* < 0.0001; Cochran‒Armitage trend test]. Nevertheless, even in patients who had had CD for ≥20 years, remission rates after 4 and 24 weeks were 28.8 % and 28.8%, respectively, by NRI, and 32.7% [32/98 patients] and 41.0% [32/78 patients], respectively, by as-observed analysis.

Remission rates were also compared between patients with and without previous anti-TNFα therapy. Anti-TNFα-naive patients had a significantly higher remission rate compared with anti-TNFα‒treated patients at 4 weeks [56.2% vs 36.7% by NRI, and 67.4% vs 40.9% by as-observed analysis; *p* < 0.0001] and 24 weeks [38.3% vs 30.6% by NRI and 68.7% vs 40.3% by as-observed analysis; *p* < 0.0001].

**Figure 4.**
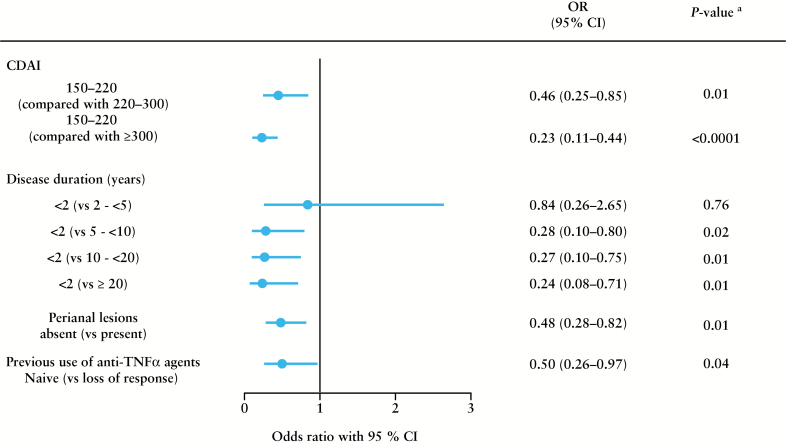
Factors affecting the effectiveness of adalimumab identified by multiple logistic regression with as-observed analysis [patients were considered responders if they showed a response at 24 weeks]. ^*a*^ *p*-value was calculated using Wald’s chi-square test. CI, confidence interval; CDAI, Crohn’s Disease Activity Index; OR, odds ratio; TNFα, tumour necrosis factor alpha.

The results of the multivariate analysis identified the following risk factors affecting effectiveness of adalimumab: CDAI > 220, disease duration >2 years, perianal lesions, and LOR to previous anti-TNFα agents [Figure 4].

## 4. Discussion

In this all-case post-marketing study of adalimumab, the safety profile was similar to that reported for previous clinical studies, ^5,8^ and no new safety concerns were noted. The most frequent serious ADR by SOC was infections [6.6 events/100 PYs].

Tuberculosis screening before anti-TNFα therapy has been recommended by the US Centers for Disease Control and Prevention, the American Thoracic Society, and the British Thoracic Society,^9^ because anti-TNFα agents may increase of the risk of tuberculosis.^10^ It has been reported that specific diseases, including IBD and rheumatoid arthritis, contribute to the increased incidence of tuberculosis. The incidence of tuberculosis in patients with IBD is about double that in control subjects.^11^ Moreover, tuberculosis is more prevalent in Asian countries than in Western countries.^12^ In Japan, the prevalence and incidence of tuberculosis are 26 and 20 per 100 000 population, respectively, according to World Health Organization data from 2011. Therefore in Japan, where tuberculosis is more common than in Western countries, a relatively large number of patients may be expected to be found to have tuberculosis despite careful screening for tuberculosis before initiating adalimumab therapy. Given the relatively high prevalence of tuberculosis in Japan and the rare incidence of active tuberculosis during the study, tuberculosis screening and the use of preventive medication can be considered appropriate measures for the prevention of tuberculosis in this population.

The Japanese guidelines for the use of biologicals recommend screening measures such as patient interviews, the tuberculin skin test, and chest X-ray for identification of previous tuberculosis infection.^13^ In the present study, one patient was found to have active tuberculosis during adalimumab therapy, despite baseline screening including chest X-ray, CT scan, and the tuberculin skin test. Although his tuberculin skin test showed a positive reaction without induration or double redness, he did not have any abnormal findings on chest X-ray or CT scan. False-positive tuberculin skin tests are more common in people with a past history of BCG vaccination, which has been common in Japan as a measure for tuberculosis prevention, ind t is difficult to detect previous tuberculosis infection by erythema size in people with a history of BCG.^14^ This case shows the importance of tuberculosis prevention; appropriate screening tests should be used to avoid the unnecessary use of isoniazid therapy, which may cause ADRs such as liver damage.^15^ IGRA has a higher specificity and sensitivity than the tuberculin skin test for the detection of reactivation of latent tuberculosis infection,^14^ so IGRA should be used before adalimumab therapy to detect latent tuberculosis infections in patients with a history of BCG vaccination.

Before the study treatment, 258 of 1693 patients [15.2%] had neither tuberculin skin test/IGRAs nor chest X-ray/CT scan. However, 240 of the 258 patients had a history of anti-TNFα therapy, and therefore might have undergone tuberculosis screening before the previous treatment. Similarly, screening for hepatitis B virus was also carefully conducted. In the present study, 289 of 1693 patients [17.1%] did not have any hepatitis B tests, but 230 of them had a history of anti-TNFα therapy for which hepatitis B screening is strongly recommended.

The risk factors for serious ADRs, as identified by the multiple logistic regression analysis, included inpatient status at baseline, being the recipient of injections by medical personnel, and concomitant use of total parenteral nutrition. Increased disease activity may have contributed to these risk factors. Concomitant use of IMs was not identified as a risk factor for serious infections. In the subpopulation of patients who had discontinued previous anti-TNFα therapy because of ADRs, only 7.2% discontinued treatment with adalimumab because of ADRs in the present study, thereby showing the high tolerability of adalimumab [Table 4].

The 24-week observation period of the present study may be short for the thorough investigation of potential safety issues. However, we consider that the length of the observation period was sufficient for the safety assessment, based on several previous reports. Kiss *et al*.^16^ reported that, in their 52-week study involving 221 patients with CD, pneumonia was reported in two patients and tuberculosis in one patient as serious infections during the first 24 weeks of treatment with adalimumab. Cordero *et al*.^17^ reported that, in their small-scale clinical study of adalimumab with a 48-week observation period, 2 of a total of 25 patients with CD had serious AEs [tuberculous meningitis and abdominal abscess] during the first 24 weeks of treatment. Moreover, although the study patients were different from those in the present study, Galloway *et al.* ^18^ reported that, in their study using data from a large-scale patient registry, the risk of serious infections in patients with rheumatoid arthritis receiving anti-TNFα therapy is most commonly noted in the first 24 weeks of treatment.

With regard to the effectiveness, > 40% of patients achieved clinical remission at 4 weeks, as shown by both as-observed and NRI analyses. At 24 weeks, the remission rate was 32.4% by NRI; this was lower than the 51.5% by as-observed analysis, which included 433 patients [after excluding 255 patients who dropped out]. Reasons for patient dropout were lack of effectiveness [44.2%], AEs [18.9%], transfer to another hospital or lack of further visits after administration [18.9%], and other reasons [18.0%].

The results of the multivariate analysis showed that CDAI > 220, disease duration > 2 years, perianal lesions, and LOR to previous anti-TNFα agents were risk factors affecting effectiveness of adalimumab. Perianal lesions have been reported to be a prognostic factor for poor response to CD treatment, and are often complicated by poor wound healing and recurrences.^19^ The effectiveness of adalimumab was higher in anti-TNFα-naive patients than in anti-TNFα‒treated patients. In addition, adalimumab was most effective in patients with short disease duration. These results were consistent with the results of the CHARM study,^20^ which showed that patients with disease duration of < 2 years were most likely to achieve remission, and that effectiveness was greater in anti-TNFα-naive patients than in anti-TNF‒exposed Japanese patients.^5^ In the present study, clinical remission rates after 4 and 24 weeks were significantly higher in patients with shorter disease duration than in those with longer duration. The results of previous studies have shown that adalimumab can be expected to be more effective in patients with short disease duration and in biological-naive CD patients.^21^

In Japan, the only anti-TNFα agents currently approved for the treatment of CD are adalimumab and infliximab. Because the present study included patients who had not been treated with adalimumab, the previous anti-TNFα agent is infliximab. It has been reported that non-responders to infliximab often do not respond to adalimumab, or they lose the effectiveness of adalimumab in a short period of time.^22^ In the present study, treatment-naive patients had a significantly higher remission rate at 4 weeks than patients with LOR to previous anti-TNFα therapy [56.2% vs 34.4% by NRI and 67.4% vs 39.2% by as-observed analysis; *p* < 0.0001] and 24 weeks [38.3% vs 26.7% by NRI and 68.7% vs 42.8% by as-observed analysis; *p* < 0.0001].

The above results were obtained for patients with baseline CDAI ≥ 150; we did the same analyses including data from patients with baseline CDAI ≥ 220. The clinical remission rate in patients with baseline CDAI ≥ 220 was slightly lower than that in patients with baseline CDAI ≥ 150 [Supplementary Figure 2, available as Supplementary data at *ECCO-JCC* online]. Nevertheless, the multivariate analysis showed a similar trend [Supplementary Figure 3, available as Supplementary data at *ECCO-JCC* online]. In the present study, of 1017 patients whose CDAI was measured before and after treatment with adalimumab, 329 patients had baseline CDAI < 150. Because of the nature of the study design [a prospective observational, non-interventional, study conducted in a daily clinical setting], baseline CDAI score was not specified as an inclusion criterion. In addition, 77.1% of patients enrolled in the present study had received previous anti-TNFα therapy and switched to adalimumab because of LOR to the former anti-TNFα agent, or in some cases because of safety issues. Therefore, patients were enrolled not only based on CDAI, and some patients had CDAI < 150.

Few previous studies evaluated combination therapy with adalimumab and IMs. In a 56-week study, adalimumab was shown to be effective both with and without concomitant use of IMs.^4^ In a systematic review of IMs used in the treatment of IBD, concomitant use of IMs with adalimumab was not associated with change in the effectiveness or dose of adalimumab,^23^ whereas another study suggested that combination of adalimumab and IMs might lead to a slight decrease in the percentage of patients who fail to respond to adalimumab and a lower need for dose escalation of adalimumab during the early stage of treatment.^24^ In the present study, there was no difference in the effectiveness of adalimumab between patients with and without concomitant use of IMs in the analyses of all patients [Supplementary Figure 1].

This study has several limitations. Because this non-interventional study was performed in the real-world clinical setting, patients were not selected according to strict criteria in terms of previous and concomitant medication other than biological drugs. In addition, because of the nature of the post-marketing study, a certain number of patients dropped out during the observational period. Moreover, the comparisons of ADRs by baseline patient characteristics may not be the most appropriate, because of imbalance in terms of baseline characteristics between patient subgroups. However, we believe that these reflect daily clinical practice when adalimumab is used.

In conclusion, the safety and effectiveness of adalimumab were confirmed in Japanese patients with CD in the clinical setting. The most frequent serious ADR was infection. The safety profile of adalimumab is consistent with previously published reports. Regarding the relatively high incidence of tuberculosis in Asian countries, the safety of adalimumab in Japan has been shown to be achievable with the use of preventive antituberculosis medication and screening tests. In patients with a history of anti-TNFα therapy and who switched to adalimumab due to ADRs, adalimumab was well tolerated.

## Funding

This work was supported by an unrestricted grant from AbbVie G.K. and Eisai.

## Conflict of Interest

H.O has received consulting fees from AbbVie G.K., Janssen Pharmaceutical K.K., and Mochida Seiyaku. MW has received grant/research support or consulting fees from AbbVie G.K., Otsuka Pharma, MSD K.J., Ajinomoto Pharma, Asahi Kasei Kuraray Medical, JIMRO, Kyorin Pharmaceutical, Tanabe Mitsubishi Seiyaku, UCB Japan, Zeria Pharmaceutical, Chugai Pharmaceutical, Astellas Pharma, and Kyowa Hakko. TM has received research funding from AbbVie G.K., Eisai, JIMRO, Zeria Pharmaceutical, Ajinomoto Pharma, Tanabe Mitsubishi Se, and Nissin Kyorin Pharmaceutical. HH, MO, TT, and YS are employees of AbbVie G.K. TH has received grant/research support or consulting fees from AbbVie G.K., Ajinomoto Pharma, Asahi Kasei Kuraray Medical, AstraZeneca Pharmaceuticals, Janssen Pharmaceutical K.K., JIMRO, Nissin Kyorin Pharmaceutical, Otsuka Pharma, Tanabe Mitsubishi Seiyaku, UCB Japan, UMN Pharma, and Zeria Pharmaceutical. HO, MW, TM, and THhave served on a safety advisory committee for AbbVie G.K.

## Author Contributions

HO, MW, TM, and TH contributed to acquisition and interpretation of data, and writing or critically reviewing the manuscript. HH, MO, TT, and YS contributed to the design, conduct, and analysis of the study, and writing or critically reviewing the manuscript. All authors approved the final version of the manuscript for submission.

## Supplementary Data

Supplementary data are available at *ECCO-JCC* online.

Supplementary Table 1a, available as Supplementary data at ECCO-JCC online
