# Supplementary material for: Safety of Adalimumab and Predictors of Adverse Events in 1693 Japanese Patients with Crohn’s Disease
Source: J Crohns Colitis. 2016 Mar 9;10(9):1033–41. doi: 10.1093/ecco-jcc/jjw060 (PMC5007524; doi:10.1093/ecco-jcc/jjw060)
Supplement: Supplementary Table 1a, available as Supplementary data at ECCO-JCC online [file ecco-jcc_jjw060_index.html]

Supplementary Data | Journal of Crohn's and Colitis

## Supplementary Data

Data files

- Supplementary Data - Supplementary Data
