# Supplementary material for: Safety of Adalimumab and Predictors of Adverse Events in 1693 Japanese Patients with Crohn’s Disease
Source: J Crohns Colitis. 2016 Mar 9;10(9):1033–41. doi: 10.1093/ecco-jcc/jjw060 (PMC5007524; doi:10.1093/ecco-jcc/jjw060)
Supplement: Supplementary Table 1a, available as Supplementary data at ECCO-JCC online [file ECCO_JCC_2015_0756_CD1_7K_PMS_Supplementary_Data.pptx]

## Slide 1
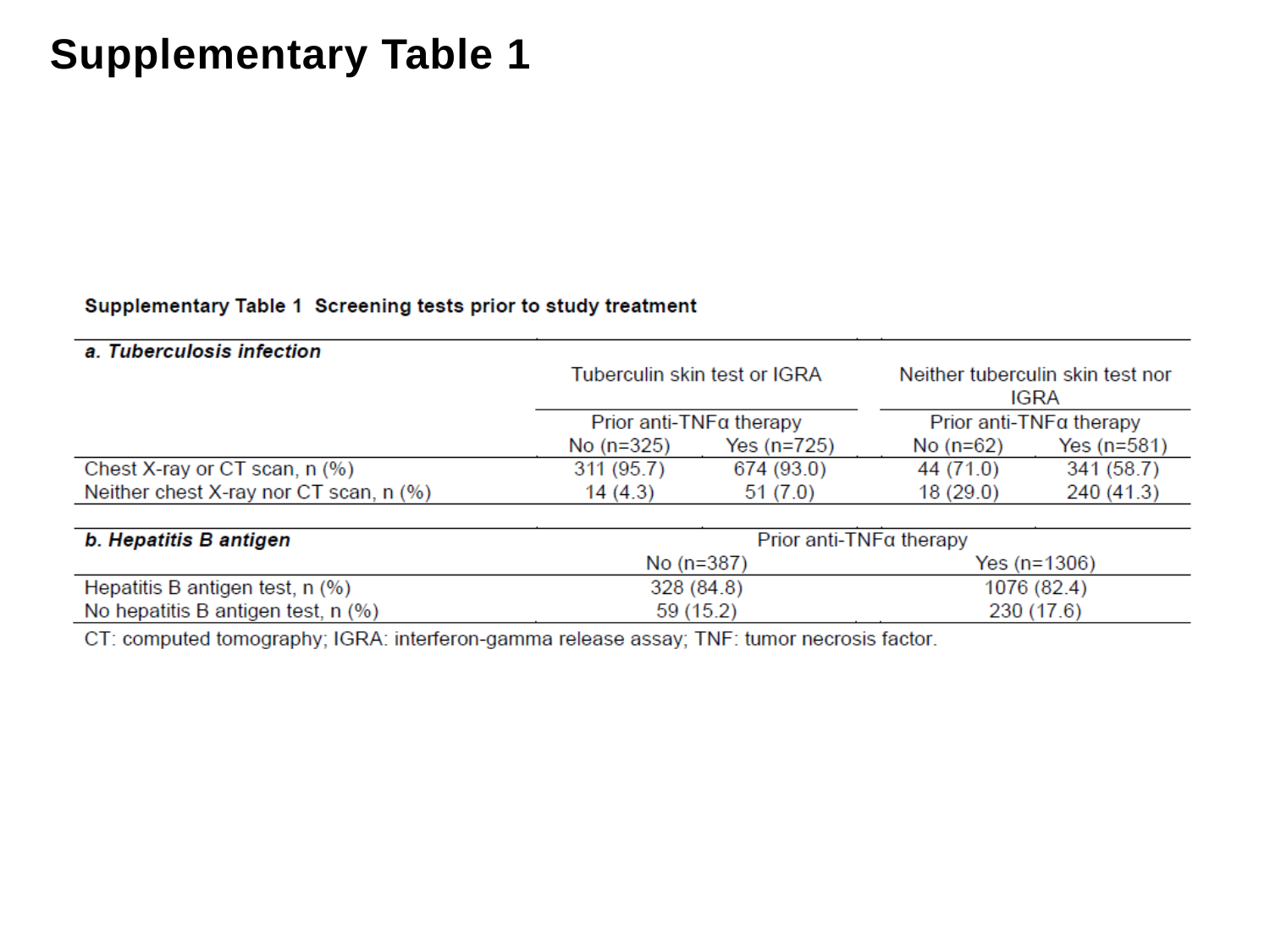

Supplementary Table 1

## Slide 2
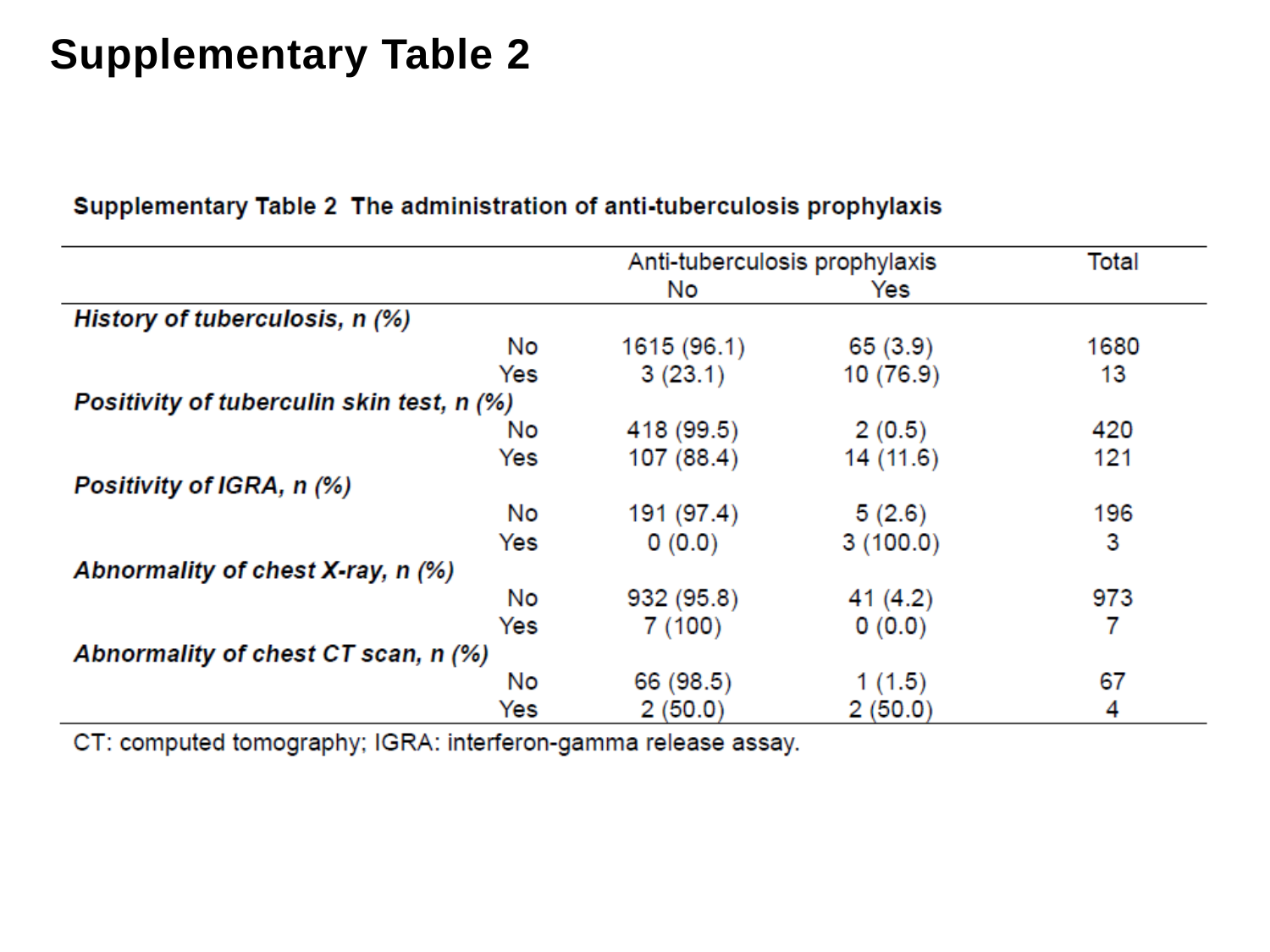

Supplementary Table 2

## Slide 3
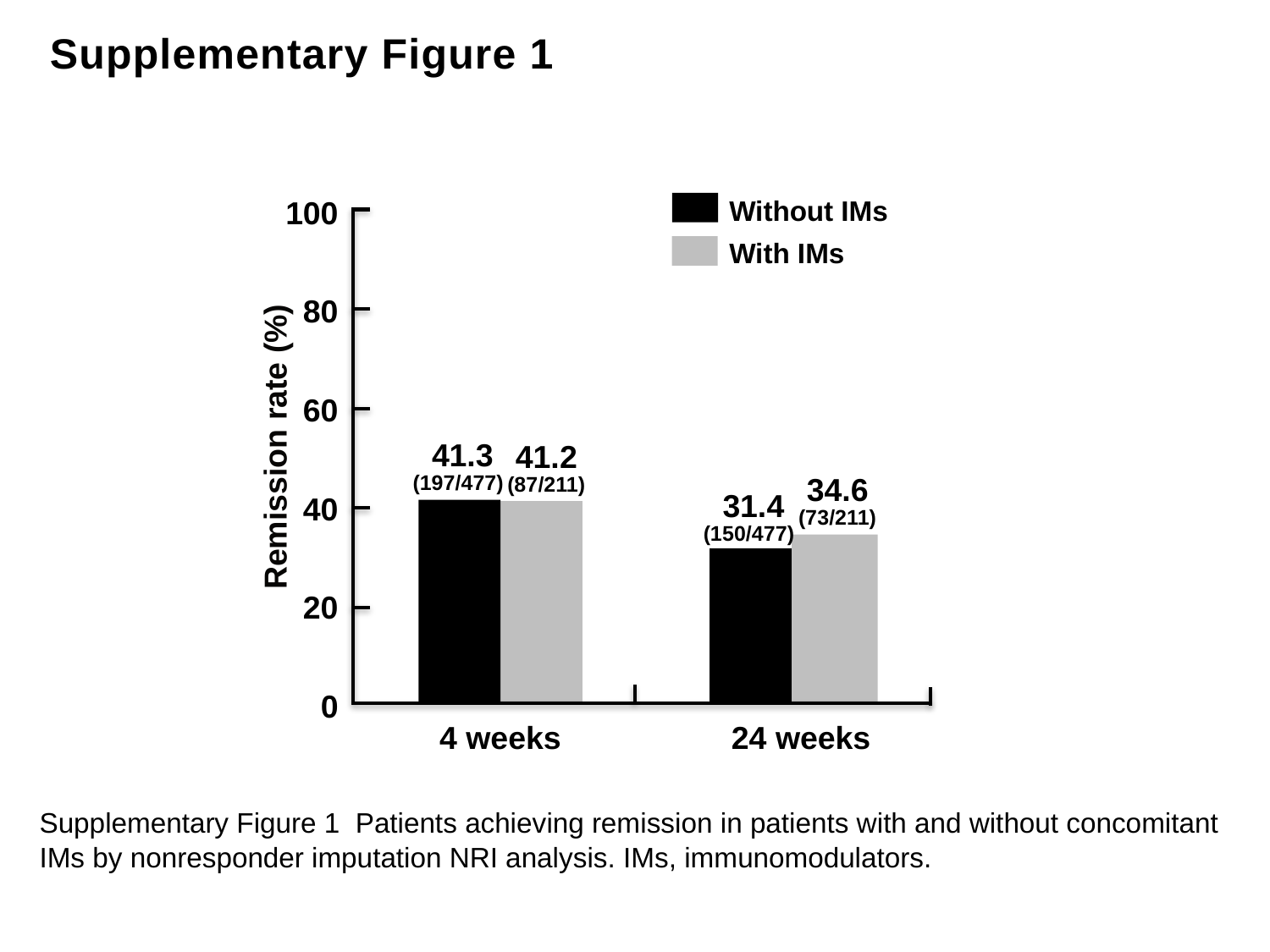

Supplementary Figure 1
Without IMs
With IMs
100
80
60
Remission rate (%)
 41.3
(197/477)
41.2
(87/211)
34.6
(73/211)
40
 31.4
(150/477)
20
0
4 weeks
24 weeks
Supplementary Figure 1 Patients achieving remission in patients with and without concomitant IMs by nonresponder imputation NRI analysis. IMs, immunomodulators.
All case of CD PMOS (CD1.7K) | Date: Sep.25, 2014| Company Confidential © 2014

## Slide 4
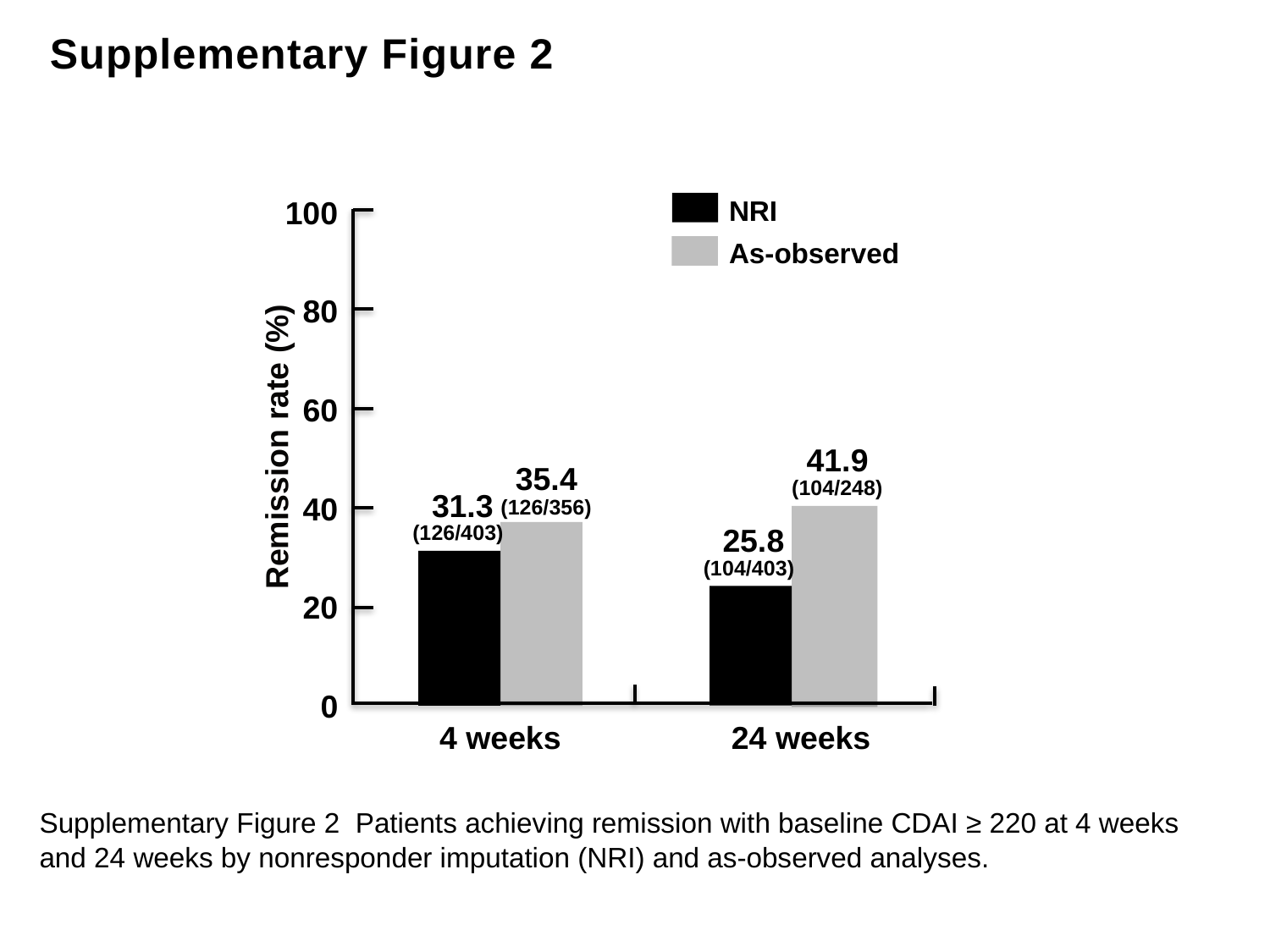

Supplementary Figure 2
100
NRI
As-observed
80
60
Remission rate (%)
41.9
(104/248)
35.4
(126/356)
40
 31.3
(126/403)
 25.8
(104/403)
20
0
4 weeks
24 weeks
Supplementary Figure 2 Patients achieving remission with baseline CDAI ≥ 220 at 4 weeks and 24 weeks by nonresponder imputation (NRI) and as-observed analyses.
4

## Slide 5
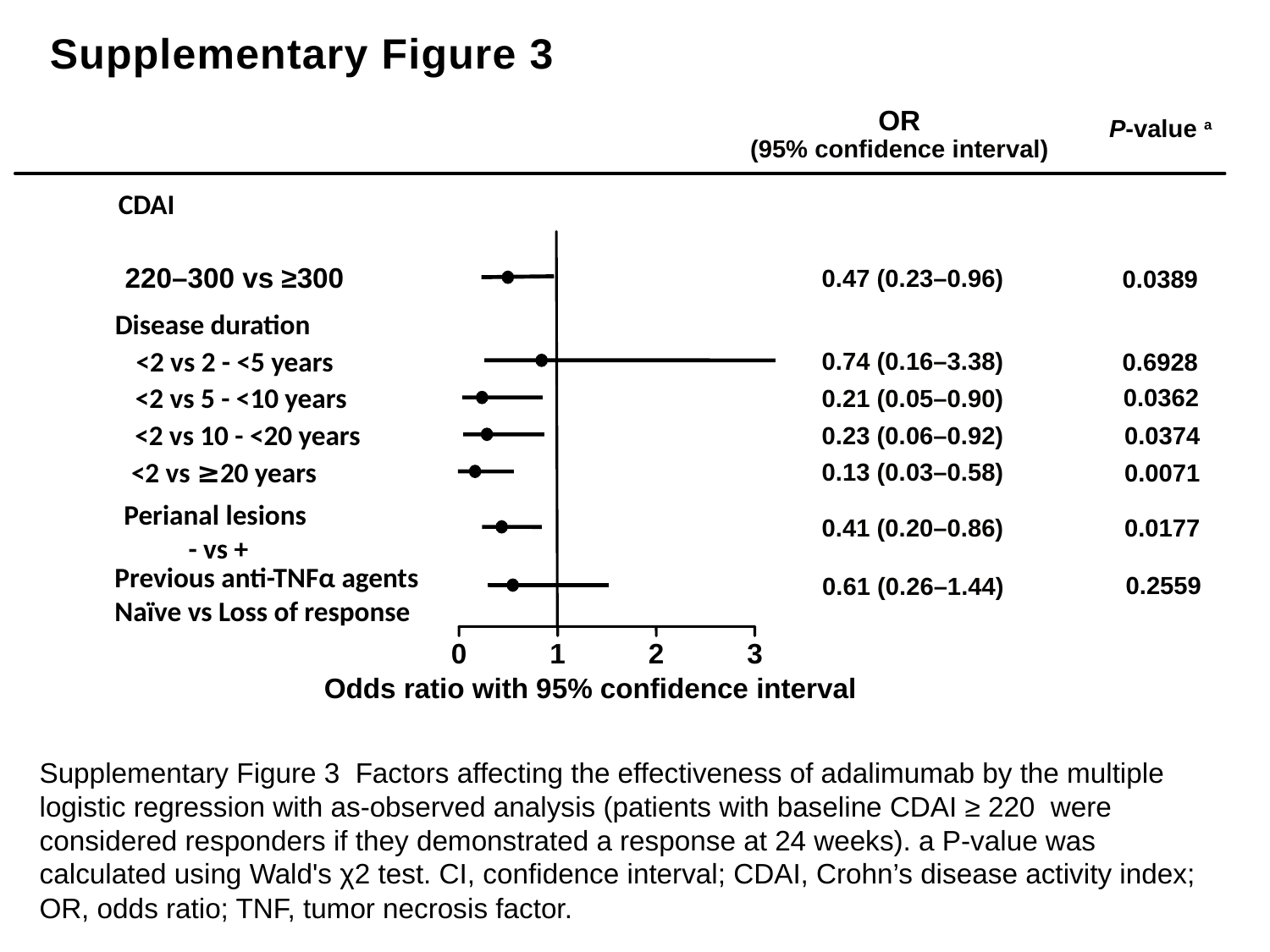

Supplementary Figure 3
OR
(95% confidence interval)
P-value a
CDAI
220–300 vs ≥300
0.0389
0.47 (0.23–0.96)
Disease duration
<2 vs 2 - <5 years
0.6928
0.74 (0.16–3.38)
<2 vs 5 - <10 years
0.0362
0.21 (0.05–0.90)
<2 vs 10 - <20 years
0.0374
0.23 (0.06–0.92)
<2 vs ≥20 years
0.0071
0.13 (0.03–0.58)
Perianal lesions
 - vs +
0.0177
0.41 (0.20–0.86)
Previous anti-TNFα agents
Naïve vs Loss of response
0.2559
0.61 (0.26–1.44)
0
1
2
3
Odds ratio with 95% confidence interval
Supplementary Figure 3 Factors affecting the effectiveness of adalimumab by the multiple logistic regression with as-observed analysis (patients with baseline CDAI ≥ 220 were considered responders if they demonstrated a response at 24 weeks). a P-value was calculated using Wald's χ2 test. CI, confidence interval; CDAI, Crohn’s disease activity index; OR, odds ratio; TNF, tumor necrosis factor.
